# Altered hypothalamic metabolism in chronic cluster headache patients measured with ^1^H-MRS at ultra-high magnetic field

**DOI:** 10.1186/s10194-026-02446-4

**Published:** 2026-07-04

**Authors:** Avneesh Jain, Anna Xiu Rørnes, Anne Farestveit, Maria Tunset Grinde, Axel Karl Gottfrid Nyman, Beathe Sitter, Tore Wergeland, Erling Tronvik, Guglielmo Genovese

**Affiliations:** 1 Norwegian Centre for Headache Research, Faculty of Medicine and Health Sciences, Norwegian University of Science and Technology, Trondheim, Norway; 2 Department of Neuromedicine and Movement Science, Faculty of Medicine and Health Sciences, Norwegian University of Science and Technology, Trondheim, Norway; 3 Faculty of Medicine and Health Sciences, Norwegian University of Science and Technology, Trondheim, Norway; 4 Department of Radiology and Nuclear Medicine, St. Olavs University hospital, Trondheim, Norway; 5 Department of Circulation and Medical Imaging, Faculty of Medicine and Health Sciences, Norwegian University of Science and Technology, Trondheim, Norway; 6 Neuroclinic, St Olav’s University Hospital, Trondheim, Norway

**Keywords:** 7 T, Glutamate, N-acetyleaspartate, Neuronal metabolism, Mitochondria, Energy metabolism, Glutamatergic neurons

## Abstract

**Background:**

The hypothalamus is considered a central hub in the pathophysiology of cluster headache (CH), yet its neurochemical profile remains poorly understood. Proton magnetic resonance spectroscopy (^1^H-MRS) offers a non-invasive tool for in vivo assessment of brain metabolites related to neuronal integrity and energy metabolism, but previous studies in CH have been restricted to conventional magnetic field scanner (≤ 3 T), which has resulted in limited metabolic information. Ultra-high field (7 T) MR scanners enhance ^1^H-MRS with improved signal-to-noise ratio and separation of metabolite signals, providing more accurate and broader metabolic information, which may refine our understanding of hypothalamic dysfunction in chronic cluster headache (cCH). This study investigated whether patients with cCH exhibit altered hypothalamic metabolite concentrations compared with healthy volunteers (HVs).

**Methods:**

^1^H-MRS at 7 T was performed in 10 cCH patients and 11 HVs to measure the neurochemical profile of the hypothalamus. Spectra were quantified with LCModel, yielding ratios of 9 metabolites relative to total creatine (tCr). Welch’s unpaired t-tests were performed as a first-level exploratory analysis to compare metabolites between the two groups. Then, group differences in metabolite ratios were examined using analysis of covariance, with age and sex included as covariates to control for potential demographic effects.

**Results:**

Compared with HVs, cCH patients showed significantly reduced glutamate (Glu)/tCr (∼17%; *p* < 0.001) and N-acetyleaspartate (NAA)/tCr (∼10%; *p* = 0.048), with no other metabolite differences. After adjusting for covariates, a significant group effect was still observed for Glu/tCr (*p* = 0.0045), whereas no group difference was found for NAA/tCr (*p* = 0.079). Tissue composition within the voxel did not differ between groups, indicating that the observed metabolic differences were unlikely to be driven by partial-volume effects.

**Conclusions:**

This is the first study to characterize hypothalamic metabolism in cCH at 7 T. The observed reductions in Glu, together with a statistically less robust decrease in NAA, indicates hypothalamic neuronal involvement, possibly reflecting mitochondrial and energetic dysfunction. This ultrahigh-field finding extends previous results at conventional magnetic field intensities, offering more specific neurochemical evidence for hypothalamic involvement in cCH.

**Supplementary Information:**

The online version contains supplementary material available at 10.1186/s10194-026-02446-4.

## Introduction

Cluster headache (CH) is a class of primary trigeminal autonomic cephalgia, which affects less than 1% of the population worldwide [1]. It is characterized by unilateral attacks of excruciating pain primarily in the orbital region as well as cranial autonomic features (conjunctive injection, rhinorrhea, nasal congestion, lacrimation and/or hyperhidrosis), ipsilateral to the attack [2]. Depending on the rate of occurrence, CH has been classified into two key forms: (1) episodic cluster headaches (eCH), where the pain occurs in bouts lasting 7 days to 1 year separated by 1 month pain-free periods and (2) chronic cluster headaches (cCH), with attack frequency for more than 1 year without remission or less than 1 month remission period [2].

Anatomical studies have observed that the projections from the pain afferent trigeminovascular neurons innervate various nuclei in the hypothalamus, thalamus and brainstem which then further projects to higher cortical areas involved in pain processing, including the frontal cortex, insula and cingulate cortex [3, 4]. These projections might play a role in parasympathetic hyperactivity mediating autonomic features and hypothalamic functions such as loss of appetite and fatigue commonly observed in CH [5]. Collectively, these findings [3, 4] suggest that neuroanatomical connections to and from the hypothalamus might play a role in CH pathophysiology. Also, further evidence of altered circadian rhythmicity and neuroendocrine signaling during the CH bout suggests the involvement of the hypothalamus, which is thought to have specific control over cyclic phenomena, in the overall pain initiation and/or transmission [6, 7].

Neuroimaging studies in CH patients have, likewise, also shown a central role of the hypothalamus both during and outside CH attacks [8]. Specifically, during CH attacks, activation of the hypothalamus was first demonstrated with positron emission tomography (PET) [9–11] and later confirmed with functional MRI (fMRI) during both spontaneous and nitroglycerin-triggered attacks [12, 13]. Outside of CH attacks, persistent hypothalamic abnormalities were revealed with resting state fMRI (rs-fMRI) [14, 15] and voxel-based morphometry (VBM) [16, 17]. Specifically, altered functional connectivity among the hypothalamus and cortical, diencephalic, and cerebellar regions were revealed with rs-fMRI [14, 15], indicating sustained neuronal dysfunction even during pain-free intervals. Also, VBM studies showed grey matter alterations in the hypothalamus of eCH and cCH, suggesting the presence of hypothalamic structural alterations in- and out-of-bout CH attacks [16, 17].

Proton magnetic resonance spectroscopy (¹H-MRS) has been proved as a valid tool to provide detailed neurochemical information in many clinical applications in vivo [18]. The quantification of metabolite concentrations from a specific volume of interest in the human brain reflects distinct pathophysiological mechanisms within that area, such as neuronal integrity and energy metabolism [19–21].

With the advent of ultra-high-field scanners (i.e., 7 T scanners) and the consequent increase of signal-to-noise ratio (SNR) and spectral dispersion, it is now possible to quantify more than 15 metabolites [22]. Specifically, in contrast to lower fields strength (i.e., ≤ 3 T) it is possible to disentangle the metabolic signal from various metabolites complexes, such as glutamate (Glu) and glutamine (Gln), and *N*-acetylaspartate (NAA) and N-acetylaspartylglutamate (NAAG). Although the chemical structures of Glu and Gln, and of NAA and NAAG, are similar and they resonate closely, resolving their signals is particularly important as their role in physiology and their cell population specificity are different [20, 21]. Indeed, NAA is generally accepted as a neuronal marker, whereas NAAG is a neuropeptide [19–21]; Glu is the main excitatory neurotransmitter of the human brain with the principal reservoir in the neurons, whereas Gln is the Glu precursor and it is mainly stored in astrocytes [20, 21].

The aim of this exploratory study was to examine the neurochemical profile of the hypothalamus in cCH. To achieve this, 7 T ¹H-MR spectra were acquired from the hypothalamus of cCH patients and compared to those from healthy volunteers (HVs). As per the current literature, just two ¹H-MRS studies have been conducted to investigate the neurometabolism of the hypothalamus in the CH patients at lower field (≤ 3 T) [23, 24]. The involvement of hypothalamic neuronal nuclei was expected as reduced NAA signals were observed in these previous works [23, 24].

## Methods

### Study population

Twelve cCH patients (age: 38 ± 11 years; age range: 22–57; 7 women) and seventeen HVs (age: 34 ± 11 years; age range: 22–57; 11 women) were recruited through a secondary neurology outpatient clinic at St Olav’s university hospital, Trondheim, Norway. Informed consent was obtained before participation. The project was approved by the Norwegian regional committee for medical and health research ethics (REK ref. 253435).

Eligibility criteria were reviewed by a consultant neurologist for all participants. Inclusion criteria for cCH patients were: (1) fulfilment of the cCH criteria according to the international Classification of Headache Disorders (ICHD-3) [2]; (2) no headache medication overuse. Patients were asked to maintain a headache attack diary two weeks prior to the scan in order to document CH attacks and any associated autonomic symptoms. However, the diary recorded the number of attacks within a time window (6:00 PM of the previous day to 6:00 PM of the current day); therefore, the exact timing of each attack relative to the MRI/MRS acquisition was not recorded. All patients were scanned during the cluster bout, with MRI/MRS acquisitions performed during the interictal period. All scans were conducted between 9:00 AM and 12:00 PM on the day of scanning.

Inclusion criteria for HVs were the absence of: (1) any other primary or secondary headache disorders, with the exception of episodic tension-type headache, and (2) any neurological and/or psychiatric conditions associated with reduced functional capacity (e.g., multiple sclerosis, Parkinson’s disease, Alzheimer’s disease). Exclusion criteria for both groups were age < 18 years and inability to comply with MR safety, including metal implants, pregnancy and self-reported claustrophobia. No restrictions were placed on the use of preventative medication during the study.

### MRI/MRS acquisition

All scans were performed on a 7 T Siemens MAGNETOM Terra scanner (Siemens Healthineers, Erlangen, Germany) using a 1-transmitter/32-receiver-channel radiofrequency head coil (Nova Medical, Wilmington, MA, USA). Each participant was scanned in one session, in which both anatomical images and spectroscopy data were acquired. Anatomical images were acquired with magnetization prepared with 2 rapid gradient echoes (MP2RAGE; echo time [TE] = 1.99 ms; repetition time [TR] = 4.3 s; inversion time [TI]_1_ = 0.84 s; TI_2_ = 2.37 s; flip angle [FA]_1_ = 5°; FA_2_ = 6°; voxel size = 0.75 mm isotropic; matrix size = 320 ⋅ 320 ⋅ 224; acceleration factor = 3; acquisition time = 8 min 50 s).

**Fig. 1:**
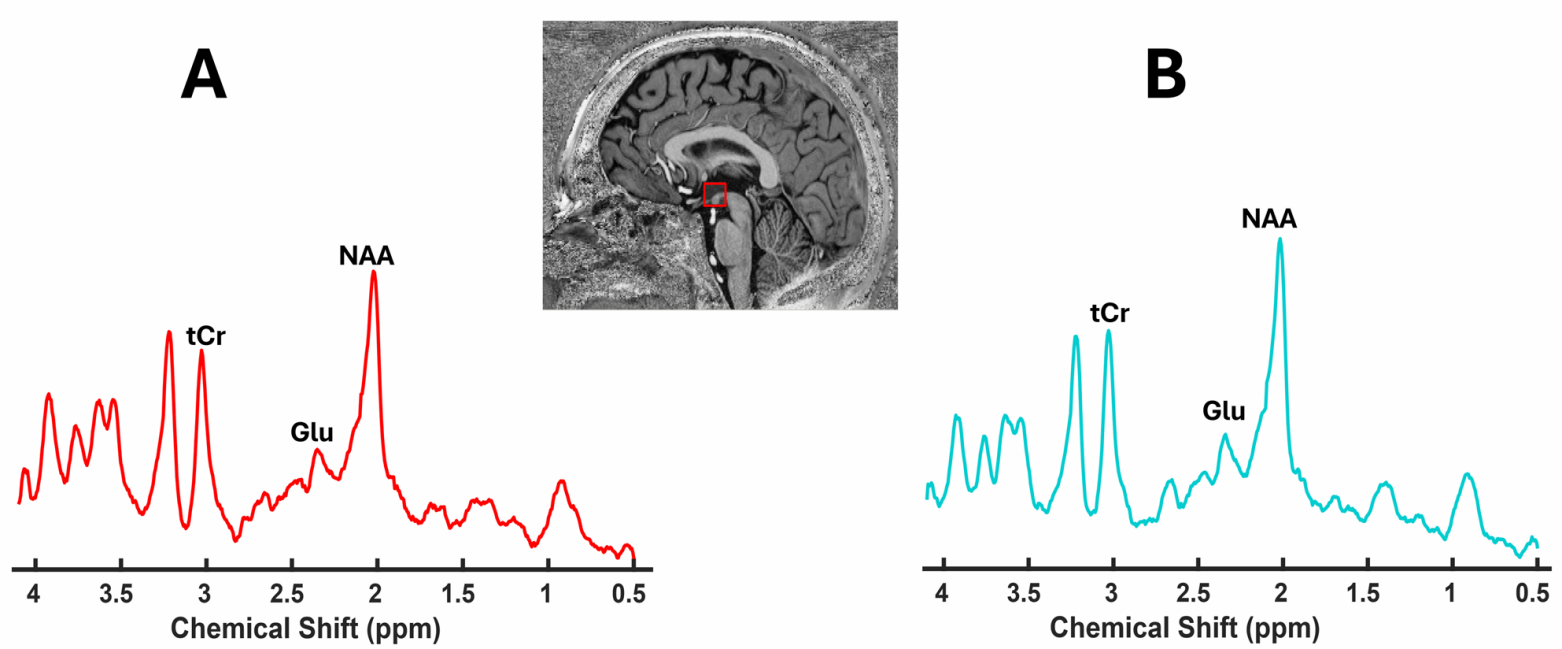
In vivo MR spectra and VOI localization. Exemplar spectrum from a cCH patient (**A**) and an HV (**B**). In vivo spectra are shown with no apodization (i.e., neither line-broadening nor zero-filling) and normalized to the signal intensity of tCr peak at 3.03 ppm. Inset: Location of the 13 mm $\:\times\:$ 13 mm $\:\times\:$ 13 mm VOI shown on the MP2RAGE image

^1^H-MRS voxels were placed in the hypothalamus (volume of interest [VOI]: 13 ⋅ 13 ⋅ 13 mm³) based on the MP2RAGE images (Fig. 1). Radiologists at St. Olavs Hospital confirmed the correct placement of the VOI for the first HVs and cCH patients. Then, a template for VOI localization on the MP2RAGE was generated in sagittal, coronal and axial views, and used for the remaining participants. Consensus on VOI localization was reached among the radiographer, MR physicist, and researcher present during the experiment for the remaining participants. First- and second- order B_0_ shimming was achieved within the VOI using an in-house procedure [25]. Briefly, all-brain B_0_ distribution maps were obtained using a 3D double echo gradient-echo sequence (TE_1_/TE_2_ = 3/4.98 ms, TR = 10 ms, FA = 7°). Then, based on the B_0_ map, shim currents were optimized to minimize the B_0_ inhomogeneities within the VOI in each subject.

The FA in the VOI was measured from a B1 map (Turbo FLASH: TE = 1.72 ms, TR = 4 s, FA = 10°) and then optimized to 90˚ by adjusting the transmit voltage. MRS data with VAPOR water suppression were collected using stimulated echo acquisition mode sequence [22] (STEAM; TE = 6 ms; TR = 5 s; mixing time [TM] = 32 ms; number of transients = 128; acquisition time = 10 min and 40 s). Signals from each receiver channel and transient were individually saved (2048 complex points, spectral width = 6 kHz) to perform spectral post-processing. Water signals were also acquired with same parameter in the VOI to perform signal coil combination. However, the water signals were not used to correct potential phase distortions in the metabolite spectra induced by eddy currents, because a systematic error affected the acquisition of the water signals.

### Spectral processing, fitting and voxel computation

Signal combination from the individual receiver channels was performed using an in-house written routine performing a weighted linear combination [26] with the coil weights identified on water signals. The coil-combined signals (i.e., 128 transients for each participant) were further processed to correct phase and frequency drifts and then averaged. Specifically, phase and frequency alignment were performed using a cross-correlation algorithm [27]. All the processing was performed in MATLAB version 2023a [28].

Spectral fitting of the averaged signals was performed using LCModel 6.3–1 N (Stephen Provencher Inc., Oakville, ON, Canada) [29]. The basis set was simulated using FID-A (an open source, MATLAB-based toolbox for simulation and processing of in-vivo ^1^H-MRS data) [30] with the following metabolites: ascorbate, aspartate, creatine (Cr), glucose (Glc), glutathione (GSH), Glu, Gln, glycerophosphorylcholine (GPC), γ-aminobutyric acid (GABA), lactate, *myo*-inositol (mIns), NAA, NAAG, phosphocholine (PCho), phosphocreatine (PCr), *scyllo*-inositol, taurine (Tau). In addition to the simulated signals, the basis set included also measured macromolecules in young adults [31]. Metabolites quantified with Cramér-Rao lower bounds (CRLBs) > 50% were excluded from the final analysis. When concentrations of two metabolites showed a strong correlation (> 0.7), as estimated by LCModel, they were summed. This was the case for Glc and Tau (Glc + Tau), GPC and PCho (tCho), and Cr and PCr (tCr). Metabolite concentrations were normalized with respect to tCr.

The SNR and linewidth (LW) of the spectra were reported as estimated by LCModel. Spectra were excluded from the analysis according to the following criteria based on the data quality: (1) SNR < 5; (2) LW > 20 Hz; (3) contamination from large water residual and/or lipids as determined by visual inspection of the spectra.

To evaluate the grey matter, white matter and cerebro spinal fluid content and the hypothalamic area within the ^1^H-MRS VOI, we performed tissue and structure segmentation using Statistical Parametric Mapping, version 12 (SPM12) [32]. First, VOI parameters defined on the MP2RAGE images (i.e., VOI dimensions along the three orthogonal VOI axes, as well as the rotation angles defining VOI orientation) were used to construct a VOI mask for each subject, separately. To address discrepancies between scanner-based coordinates and SPM12-defined MNI coordinates, subject-specific MP2RAGE images were co-registered from native scanner space to MNI space, enabling accurate overlay of the VOI mask. To ensure correct VOI placement, the registered images and mask were visually inspected in FSLeyes [33]. Finally, the tissue content of the VOI and total hypothalamic area covered were calculated using SPM12 with custom written script in MATLAB.

### Statistical analysis

Welch’s unpaired t-tests were performed as first level exploratory analysis to provide unadjusted descriptive comparisons for metabolites between cCH patients and HVs. Independent of the first level results, group differences in metabolite ratios were then examined in second level using analysis of covariance (ANCOVA), with group (cCH vs. HV) as the fixed factor and age and sex included as covariates to control for potential demographic effects. Assumptions of normality, homogeneity of variances, and homogeneity of regression slopes were verified prior to analysis. The analysis was adjusted for multiple comparisons using false discovery rate (FDR). Finally, a post-hoc power analysis based on ANCOVA’s observed effect sizes (Cohen’s f) [34] was conducted to estimate the sample size required to detect group differences in metabolites. Statistical analyses were performed using Python version 3.12.3, with statistical significance set at *p* < 0.05.

## Results

### Participant characteristics

Demographic and clinical information of cCH group is summarized in Table 1. Two cCH patients and six HVs were excluded from the analysis because the spectra did not fulfill the criteria of acceptable data quality. Then, ^1^H-MRS data from 10 cCH patients (age: 38 ± 11 years; age range: 22–57; 7 women) and 11 HVs (age: 34 ± 11 years; age range: 22–57; 6 women) were analyzed. No significant demographic differences were observed between the groups (*p* = 0.50 for age; *p* = 0.66 for gender). Based on the diary entries, all cCH participants reported at least one attack either on the day of the scan or on the preceding day.

**Table 1:** Demographic and clinical characteristics of all subjects. eTTH = episodic tension-type headache, cTTH = chronic tension-type headache, S = Sumatriptan, O₂ = Oxygen therapy, m = Maxalt, Z = Zolmitriptan, V = Verapamil, T = Topiramate, G = Galcanezumab, B = Bupivacaine, G-b = GON-blockade, K = Ketobemidone, A = Amitriptyline, M = Melatonin, L = Levetiracetam, D = Diazepam, F = Fremanezumab. *(*^X^*subjects excluded from the analysis because not acceptable*^*1*^H-MRS data quality;–*no data; **every 3 months; ***regularly)

ID	Group		Age/Sex	Daily attack frequency (14 days prior to scan)	Headache or other disorders (other than cCH)	Therapy	
						Acute	Preventive
1	cCH	48/M		6	None	None	V
2ˣ	cCH	57/F		4	None	S	V, T
3	cCH	34/M		2	None	S, O2	G, B, V
4	cCH	30/F		1	None	None	G-b**, V, K
5ˣ	cCH	48/M		2	Fibromyalgia	m	G-b***, A
6	cCH	36/F		– *	eTTH	S, O2, Z	V, T, M
7	cCH	48/F		2	cTTH	S	V
8	cCH	45/F		4	None	S	L, M
9	cCH	56/M		2	None	S	V, S
10	cCH	35/F		– *	Epilepsy	S	V, L, D
11	cCH	24/F		6	eTTH	S, O2	V
12	cCH	22/F		4	eTTH	S, O2	F
13ˣ	HV	30/M			None	None	None
14ˣ	HV	29/F			None	None	None
15	HV	23/F			None	None	None
16	HV	57/M			None	None	None
17	HV	22/F			eTTH	None	None
18	HV	25/F			None	None	None
19ˣ	HV	56/F			eTTH	None	None
20	HV	46/M			None	None	None
21	HV	36/M			None	None	None
22	HV	26/F			None	None	None
23	HV	33/F			None	None	None
24ˣ	HV	49/F			None	None	None
25	HV	27/F			None	None	None
26ˣ	HV	30/F			None	None	None
27ˣ	HV	45/F			None	None	None
28	HV	44/M			None	None	None
29	HV	40/M			None	None	None

### ^1^H-MRS Data

Spectra from both groups exhibited similar SNR (Fig. 1; Table 2). Significant higher LW in HVs with respect to those from cCH patients was observed (Table 2). Tissue content and hypothalamic area in the VOI was not significantly different between the two groups (Table 2). LCModel quantified the ratio concentration of 9 metabolites with mean CRLB < 50% (Table 2). Also, metabolite CRLBs were fairly similar between cCH patients and HVs (Table 2). NAAG data from a cCH patient was removed from the analysis due to CRLB > 50%.

**Table 2:** Neurochemical concentrations in cCH and HV. Mean values ± standard deviations and Cramér Rao Lower Bounds (CRLB) are reported for metabolites, along with SNR and LW. Tissue fraction: grey matter (GM), white matter (WM), cerebro-spinal fluid (CSF) and total hypothalamus within the VOI (HT) are also reported for each group. The indicated p-values refer to the differences in metabolite concentrations between the two groups. (*Data for one subject excluded due to CRLB > 50% of the mean)

Metabolites	Conc. cCH	CRLB cCH	Conc. HV	CRLB HV		*p*-value
	(/tCr)	(%)	(/tCr)	(%)		Conc. cCH vs. HV
	(*n* = 11)		(*n* = 12)			
GABA	0.31 ± 0.05	17.2 ± 3.4	0.37 ± 0.09	17.5 ± 5.4		0.097
Gln	0.32 ± 0.08	18.2 ± 6.4	0.41 ± 0.08	19.3 ± 4.5	0.16	
Glu	**0.94 ± 0.04**	**6.8 ± 1.3**	**1.10 ± 0.07**	**7.2 ± 1.6**	**< 0.001**	
GSH	0.19 ± 0.05	18.6 ± 6.6	0.18 ± 0.05	20.6 ± 8.5	0.52	
mIns	1.55 ± 0.14	3.7 ± 0.7	1.66 ± 0.19	3.9 ± 0.5	0.14	
NAA	**1.15 ± 0.12**	**4.2 ± 1.2**	**1.30 ± 0.11**	**4.6 ± 0.8**	**0.048**	
NAAG	0.22 ± 0.09	15.7 ± 4.3*	0.30 ± 0.08	16 ± 4.5	0.074	
tCho	0.33 ± 0.03	6.1 ± 1.5	0.36 ± 0.04	6.3 ± 1.6	0.10	
Glc + Tau	0.53 ± 0.16	14.7 ± 5.1	0.64 ± 0.24	12.6 ± 3.2	0.30	
SNR	12 ± 3		11 ± 3		0.59	
LW (Hz)	**12.0 ± 1.3**		**14.0 ± 2.5**		**0.037**	
GM (%)	39.6 ± 5.9		38.8 ± 6.6		0.78	
WM (%)	40.4 ± 6.3		35.4 ± 8.0		0.11	
CSF (%)	19.8 ± 8.2		25.5 ± 10.8		0.15	
HT (%)	55.0 ± 7.9		59.0 ± 15.3		0.42	

**Fig. 2:**
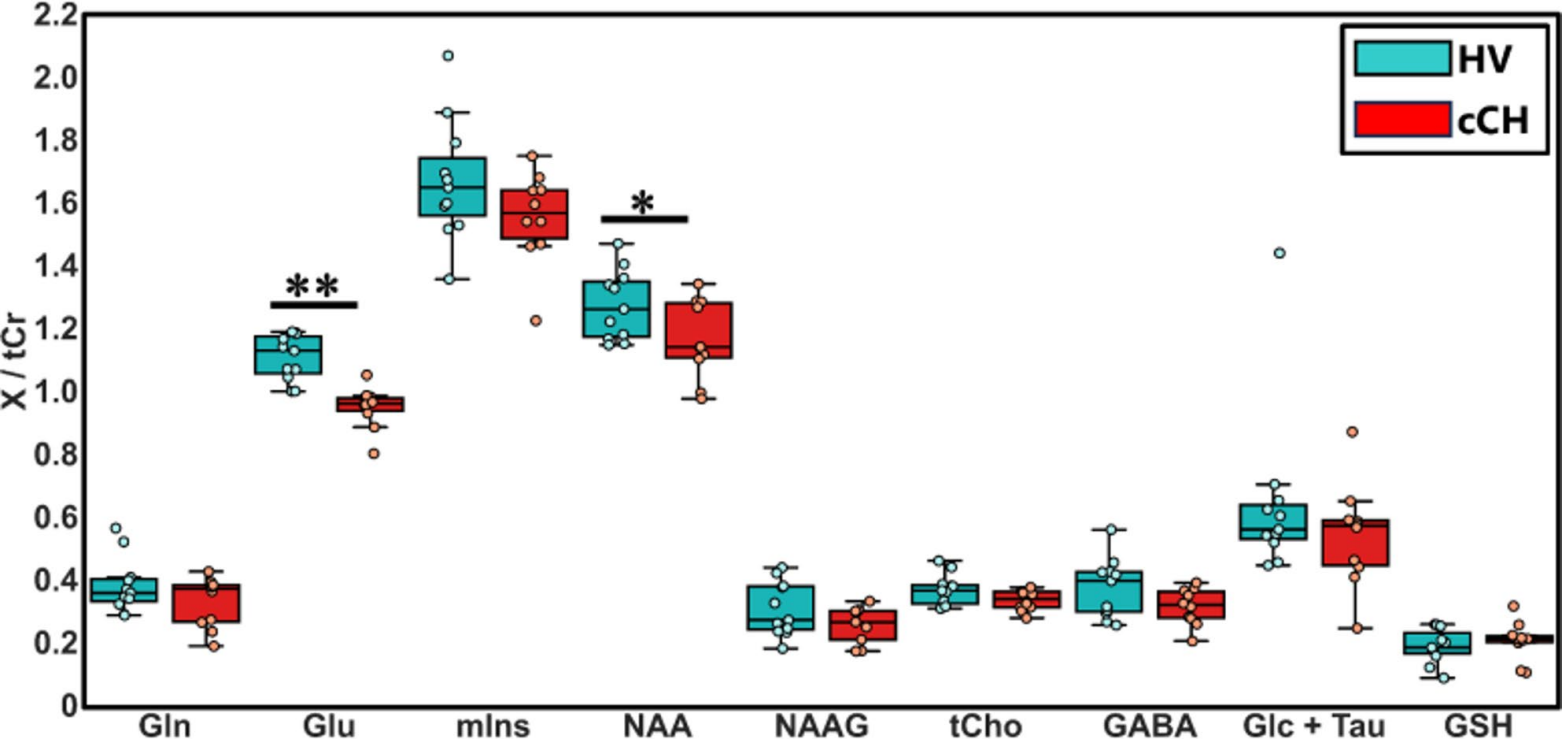
Metabolite ratio concentrations. Box plots of ratio concentrations (with respect to tCr) from HVs and cCH patients are plotted in turquoise and red, respectively. In each box, the mid-line indicates median value, and the bottom and top edges indicate the 25th and 75th percentiles, respectively. Asterisk indicates significant changes (*p* < 0.001**; *p* = 0.048*) and X indicates the concentration of a specific metabolite. (Data for NAAG of one subject excluded due to CRLB > 50% of the mean)

Significant decrease in Glu/tCr (16.7%; *p* < 0.001; 95% confidence interval (CI) = [0.09, 0.22]) was observed in cCH patients with respect to HVs (Fig. 2; Table 2). Also, NAA/tCr was significantly lower in cCH patients compared to HVs (9.4%, *p* = 0.048; 95% CI = [0.001, 0.219]; Fig. 2; Table 2). No other metabolites showed significant changes.

**Fig. 3:**
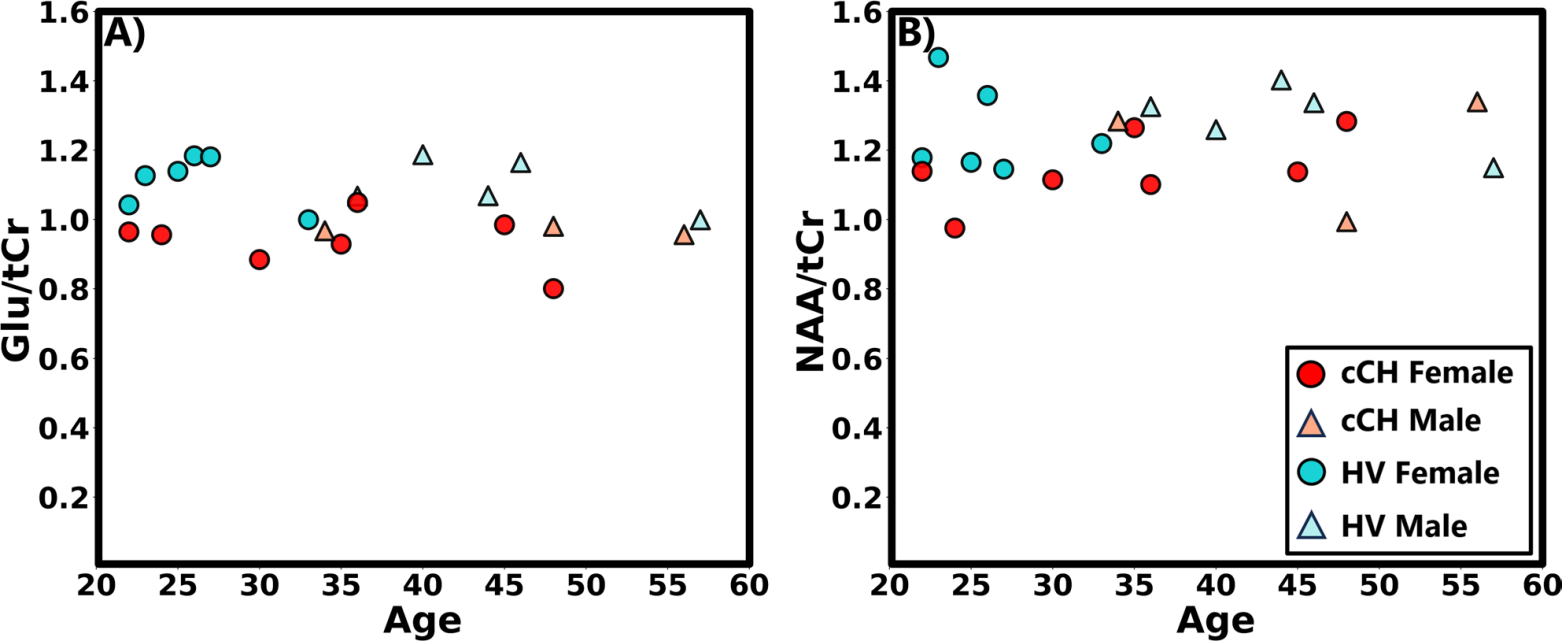
Age and sex distribution of Glu/tCr and NAA/tCr ratios in cCH patients and HVs. Scatter plots of Glu/tCr (**A**) and NAA/tCr (**B**) as a function of age. Red symbols indicate cCH patients and cyan symbols indicate HVs; circles denote females and triangles denote males. Age and sex were included as covariates in the ANCOVA analysis, which showed a significant group effect for Glu/tCr (*p* = 0.0045), but not for NAA/tCr (*p* = 0.079)

After adjusting for age and sex, a significant group effect was observed for Glu/tCr, with lower levels in cCH compared to HV (β = − 0.14, 95% CI = [− 0.21, − 0.07], FDR-adjusted p value = 0.0045; Table 3). In contrast, no significant group difference was found for NAA/tCr (β = − 0.11, 95% CI = [− 0.23, 0.01], FDR-adjusted p-value = 0.079; Table 3), as shown in Fig. 3A and B, respectively. A post-hoc power analysis based on the observed effect sized obtained after adjusting for age and sex indicated that the current sample size was sufficient to detect differences in Glu/tCr (Cohen’s f = 1.01; required *n* = 11 per group). In contrast, for NAA/tCr, a medium effect size was observed (Cohen’s f = 0.44), corresponding to an estimated requirement of approximately 43 participants per group to achieve adequate statistical power.

**Table 3:** ANCOVA analysis results. ANCOVA was performed to evaluate group differences in metabolite concentrations while accounting for the effects of age and sex across all subjects (*N* = 21). β values with 95% confidence intervals represent the estimated group effect (cCH vs. HV). F-statistics and associated p-values are reported for the group effect as well as for the contributions of age (F1) and sex (F2). The statistics for age and sex represent the variance explained by each covariate after controlling for the other factors in the model

Metabolite	Group effect			Age		Sex	
*N* = 21	β *[95% CI]*	*F*	*p-value*	*F1*	*p-value*	*F2*	*p-value*
GABA	-0.05 [-0.131, 0.029]	1.80	0.20	0.01	0.90	0.96	0.34
Glc + Tau	-0.061 [-0.293, 0.17]	0.31	0.59	0.47	0.50	1.51	0.23
Gln	-0.062 [-0.15, 0.026]	2.21	0.16	0.05	0.81	0.50	0.48
Glu	-0.140 [-0.209,0.071]	18.35	**0.0045**	2.36	0.14	1.39	0.25
GSH	0.016 [-0.041, 0.074]	0.36	0.56	0.36	0.55	0.33	0.57
mIns	-0.07 [-0.251, 0.099]	0.85	0.37	1.29	0.27	1.21	0.28
NAA	-0.108 [-0.229, 0.014]	3.48	0.079	0.06	0.80	0.20	0.66
NAAG	-0.041 [-0.123, 0.042]	1.07	0.31	5.35	0.30	3.84	0.066
tCho	-0.017 [-0.056, 0.022]	0.83	0.37	2.34	0.14	6.25	0.20

Data distributions for Glu/tCr and NAA/tCr with respect to preventive and acute treatments, comorbidities, and daily attack frequency are reported in the Supplementary Materials, as all of these factors may act as potential confounders of the findings.

## Discussion

This study aimed to characterize the neurometabolic profile of the hypothalamus in patients with cCH using ultrahigh-field MRI, and to compare it with the corresponding profile observed in HVs. This was achieved by acquiring ^1^H-MRS spectra at 7 T in 10 cCH patients and 11 HVs. To the best of our knowledge, this is the first study to measure brain metabolism in cCH at ultra-high field.

In contrast with previous work on CH performed at lower field (≤ 3 T) [23, 24] that were able to quantify only a limited number of metabolites, here the evaluation of the ratio concentrations of 9 metabolites relative to tCr was achieved. Moreover, with the increased SNR and frequency dispersion achieved at 7 T, the signals from Glu and Gln, and from NAA and NAAG were resolved and their ratio concentration were reported independently.

The primary finding of this work was the significant decrease in Glu/tCr in the cCH patients, which is consistent with the presence of pathological mechanisms affecting the neuronal cell population, as previously suggested [23, 24]. Since Glu is primarily located in neurons, decrease in Glu concentration is usually associated with excitotoxic Glu depletion [35] and/or loss of glutamatergic neurons [36, 37].

Although the presence of neurodegenerative processes remains a possible interpretation of the decreased Glu in cCH, alternative line of evidence does not suggest neuronal loss. Specifically, structural neuroimaging studies in CH performed using VBM have not revealed hypothalamic atrophy consistent with neurodegenerative processes [16, 17, 38, 39]. Even the few studies reporting hypothalamic structural alterations observed higher hypothalamic gray matter volumes [16, 17], findings more consistent with neuroplastic changes rather than tissue loss [40]. In addition, this current study shows no observed differences in the tissue content, and in the hypothalamic area in the VOIs from cCH patients with respect to those from HVs. Finally, from a clinical perspective, CH seems unlikely to relate to neurodegenerative processes, as CH patients can change phenotype over the course of disease, including remitting from the chronic to the episodic subtype [41, 42] as well as prolonged remission [43].

An alternative interpretation for reduced Glu could be altered neuronal mitochondrial function and impaired energy metabolism within the cCH hypothalamus. Glu is a central tricarboxylic acid (TCA) cycle intermediate, as it exchanges with α-ketoglutarate, and its metabolic pool is tightly linked to mitochondrial adenosine triphosphate (ATP) production, as neuronal TCA-cycle rates increase in response to energetic demand and glutamatergic activity [20, 44]. Thus, when mitochondrial oxidative phosphorylation is compromised, Glu could decline [44], reflecting impaired TCA-cycle flux and bioenergetic failure within neurons. This interpretation seems to be consistent with previous functional neuroimaging findings showing abnormal hypothalamic activation in CH patients [9, 12, 13], as synaptic activity is tightly coupled to oxidative metabolism and TCA-cycle rate [45].

However, even if altered neuronal mitochondrial function and impaired energy metabolism might represent a plausible interpretation for the reduced Glu, this cannot be confirmed from the data alone of this cross-sectional study. Also, reduced Glu is not specific to cCH and has also been reported in the interictal phase of migraine [46] and in other pain conditions [47]. Therefore, the observed Glu alterations may reflect a broader pathophysiological mechanism related to chronic pain processing. Since the present study did not include a positive control group with another chronic pain condition, the specificity of the findings to cCH mechanisms cannot be determined.

The observed decrease in NAA/tCr before correction for age and sex is consistent with previous findings at lower field [23, 24]. Similarly to the Glu/tCr decrease, it suggests the involvement of altered neuronal metabolism. Since NAA synthesis occurs mainly in neuronal mitochondria and depends directly on the availability of acetyl-coenzyme A (acetyl-CoA) [19, 20, 44], reduced NAA could reflect impaired neuronal energetics and mitochondrial dysfunction. However, reduced NAA may also reflect altered neuronal integrity or neuronal dysfunction [19–21]. Therefore, the NAA decrease, as well as Glu decrease, observed in cCH should be interpreted cautiously, as the present findings do not allow a clear distinction between neuronal metabolic impairment and neuronal loss mechanisms.

Age and sex between groups are potentially cofounders, as neurometabolic baselines [48, 49] and pathological responses differ across age and sex. After adjusting for these variables, Glu/tCr levels were significantly lower in cCH compared to healthy controls, ensuring altered glutamatergic neurotransmission represents a disease-related neurochemical feature rather than a demographic effect. NAA/tCr did not show a significant group difference suggesting that demographic factors may partially contribute to variability in NAA levels. Notably, previous studies reporting reduced NAA levels in cCH did not correct for age and sex despite using cohorts of comparable size [23, 24]. Furthermore, it should be noted that the present study was adequately powered to detect differences in Glu/tCr (*n* = 11 per group), whereas the detection of NAA/tCr differences would require a substantially larger sample (*n* = 43 per group). Therefore, the observed decreased in NAA/tCr in cCH needs to be confirmed in a larger cohort.

### Limitations and future directions

This study has several limitations that need to be acknowledged. Due to a systematic error affecting the acquisitions of the water signals, it was not possible to correct the metabolite spectra for the phase distortions induced by the eddy current. Nevertheless, the impact on spectral quality was minimal, with only slight residual phase distortion visible near 1.9 ppm (Fig. 1).

Also, the water signals were not usable as internal reference in LCModel for the metabolite concentration quantification, and then, the ratio concentrations with respect to tCr signal were reported. Although tCr is widely accepted by the MRS community as internal reference for metabolite quantification [50], the assumption of stable tCr across groups remains a potential limitation. However, if tCr were altered in cCH, a more generalized shift across all the metabolite ratios would be expected, whereas only two out of nine metabolites showed significant changes.

The higher spectral LWs observed in HVs with respect to cCH patients could also act as a confounder, since LCModel overestimates the metabolite concentrations with larger linewidths [51, 52]. Although a precise estimation of the LW influence on our data is challenging, based on previous published report [51], LW-related increase should account for at most 3%, which is substantially smaller than the > 10% differences in cCH patients with respect HVs observed here. Also, assuming uniform overestimation among metabolites, ratio-based quantification should have elided the overestimation.

Although the size of the VOI used in this study was relatively small, the VOI contained ∼ 20% of other brain regions than the hypothalamus that contribute to the metabolite signals (signal contribution from metabolites in the cerebrospinal fluid are negligible, except for Glc [53]). Therefore, some of the findings could potentially reflect metabolic alterations in adjacent mid-brain areas. However, in our data, there were no significant differences between groups in the percentage of hypothalamic area and of the tissue content within the VOI. Moreover, partial-volume effects due to the cerebrospinal fluid were further reduced because metabolite concentrations were expressed as ratios relative to tCr [53]. Taken together, these observations suggest that the observed metabolic alterations are unlikely to be explained by systematic biases related to partial-volume effects. Since only a single VOI encompassing the hypothalamic area was investigated, further investigation is required to confirm whether these metabolic alterations are specific to the hypothalamus. Notably, a previous study in CH performed with phosphorus MR spectroscopy [54] found metabolic changes in the occipital lobe potentially linked with mitochondrial impairment.

The size of the cohort in this study was relatively small, which limits the generalizability of the results and did not allow for more detailed statistical analyses accounting for comorbidities, preventive and acute treatments, and daily attack frequency. The daily attack frequency may represent a potential confounding factor in the interpretation of the neurometabolic findings, as it may reflect both disease burden and the efficacy of preventive treatments, further contributing to the clinical heterogeneity of the cCH cohort. However, the distributions of Glu/tCr and NAA/tCr values with respect to these potential confounders are reported in the Supplementary Materials.

Five out of ten patients included in the analysis had comorbidities, including episodic and chronic tension-type headache, and epilepsy, and such comorbidities may introduce additional heterogeneity in the data. However, there is currently limited evidence that tension-type headaches are associated with alterations in neurometabolism [55, 56]. Also, although epilepsy has been shown to affect the metabolic profile [57, 58], neither Glu/tCr nor NAA/tCr data from the one patient with epilepsy as comorbidity differed from the distribution of the remaining participants. Finally, the prevalence of comorbidities in our cCH cohort is consistent with previous epidemiological data [59, 60].

Furthermore, all cCH patients were receiving, in line with previous neuroimaging studies [15, 17, 24, 36, 37], preventive and/or acute headache treatments at the time of scanning, which could potentially influence neurometabolic measurements. Specifically, eight out of ten patients included in the analysis were treated with verapamil, a calcium-channel blocker [61] that may influence glutamatergic neurotransmission by reducing presynaptic calcium influx and activity-dependent Glu release. However, Glu signal detected by ^1^H-MRS predominantly reflects the large intracellular metabolic pool of Glu rather than the much smaller synaptic vesicular pool involved in neurotransmission [62] which is also largely MR-invisible [63].Therefore, pharmacological modulation of synaptic Glu release is unlikely to substantially alter the total Glu concentration detectable by MRS. Moreover, verapamil has been reported to restore astrocytic Glc metabolism and mitochondrial function in a rodent model of neurodegeneration [54], suggesting potential neuroprotective or metabolic-support effects rather than reductions in neuronal metabolites.

Also, eight out of ten patients included in the analysis were acutely treated with sumatriptan, which acts by inducing arterial vasoconstriction [64]. As triptans have limited penetration across the blood-brain barrier [64] and primarily act on extracerebral vessels [65] are also unlikely to explain the observed differences. Indeed, in a previous study combining ^1^H-MRS and MR angiography [66], metabolic signals remained unchanged despite measurable alterations in the circumferences of intra- and extracranial arteries [67].

Another limitation of this study is that the exact timing of CH attacks relative to the MRI/MRS acquisition was not recorded. Although patients reported attacks in the diary entries corresponding to the day of the scan and/or the preceding day, the diary format did not allow precise determination of whether an attack occurred within the 24 h prior to scanning. Therefore, although the present findings likely reflect baseline neurochemical changes rather than acute attack-related fluctuations, the latter confounder cannot be ruled out. Finally, since this was a cross-sectional study, causal inference cannot be made reliably.

Despite these limitations, the present findings provide preliminary insights into hypothalamic neurometabolism in cCH. This dataset reflects the application of an advanced, yet technically challenging, ultrahigh-field ^1^H-MRS approach in a relatively rare clinical population. However, the interpretation of these results should be approached with caution, as several potential confounding factors (such as comorbidities, ongoing treatments, and variations in attack frequency) could not be fully controlled in the present study. As an exploratory investigation, these results may help inform future studies including larger cohorts, multiple CH phenotypes, a positive control group, longitudinal designs, and optimized acquisition protocols, with particular attention to controlling or minimizing potential confounders.

## Conclusion

This was the first 7 T based ¹H-MRS study performed on cCH. The observed reduction in Glu, together with a statistically less robust decrease in NAA, supports the involvement of hypothalamic neuronal nuclei in cCH pathophysiology. Among the possible interpretations, mitochondrial dysfunction and impaired neuroenergetics represent a plausible mechanism for the observed alterations, though the present data do not allow this to be confirmed or distinguished from other pathological processes. In addition to providing novel insights, our findings replicate and refine the results previously obtained at lower field strengths, benefitting from the increased specificity and spectral resolution of 7 T. Future studies addressing the limitation outlined above are needed to confirm these findings and further elucidate the neurobiological mechanisms underlying cCH.

## Supplementary Information

Below is the link to the electronic supplementary material.

Supplementary Material 1

## Data Availability

Access to the datasets generated and analyzed during the current study may be granted upon reasonable request to the corresponding author and subject to compliance with applicable ethical and legal requirements.

## Abbreviations

CH: Cluster headache
eCH: Episodic cluster headache
cCH: Chronic cluster headache
PET: Positron emission tomography
fMRI: Functional magnetic resonance imaging
rs-fMRI: Resting state fMRI
VBM: Voxel–based morphometry
¹H: MRS–Proton magnetic resonance spectroscopy
SNR: Signal–to–noise ratio
Glu: Glutamate
Gln: Glutamine
NAA: N-acetylaspartate
NAAG: N-acetylaspartylglutamate
HV: Healthy volunteer
REK: Norwegian regional committee for medical and health research ethics
ICHD-3: International classification of headache disorders
MP2RAGE: Magnetization prepared with 2 rapid gradient echoes
TE: Echo time
TR: Repetition time
TI: Inversion time
FA: Flip angle
VOI: Volume of interest
STEAM: Stimulated echo acquisition mode sequence
Cr: Creatine
GABA: γ-aminobutyric acid
Glc: Glucose
GSH: Glutathione
GPC: Glycerophosphorylcholine
mIns: myo-inositol
PCr: Phosphocreatine
PCho: Phosphorylcholine
PE: Phosphorylethanolamine
Tau: Taurine
CRLB: Cramér-Rao lower bounds
tCho: GPC + PCho
tCr: Cr + PCr
LW: Linewidth
SPM12: Statistical parametric mapping, version 12
ANCOVA: Analysis of covariance
FDR: False discovery rate
CI: Confidence interval
TCA: Tricarboxylic acid
ATP: Adenosine triphosphate
acetyl-CoA: Acetyl-coenzyme A
