# Supplementary material for: Altered hypothalamic metabolism in chronic cluster headache patients measured with 1H-MRS at ultra-high magnetic field
Source: J Headache Pain. 2026 Jul 4;27(1):172. doi: 10.1186/s10194-026-02446-4 (PMC13339285; doi:10.1186/s10194-026-02446-4)
Supplement: Supplementary file 1 — Supplementary Material 1 [file 10194_2026_2446_MOESM1_ESM.docx]

**Altered hypothalamic metabolism in chronic cluster headaches patients measured with ^1^H-MRS at ultra-high magnetic field.**

**Supplementary Figures**


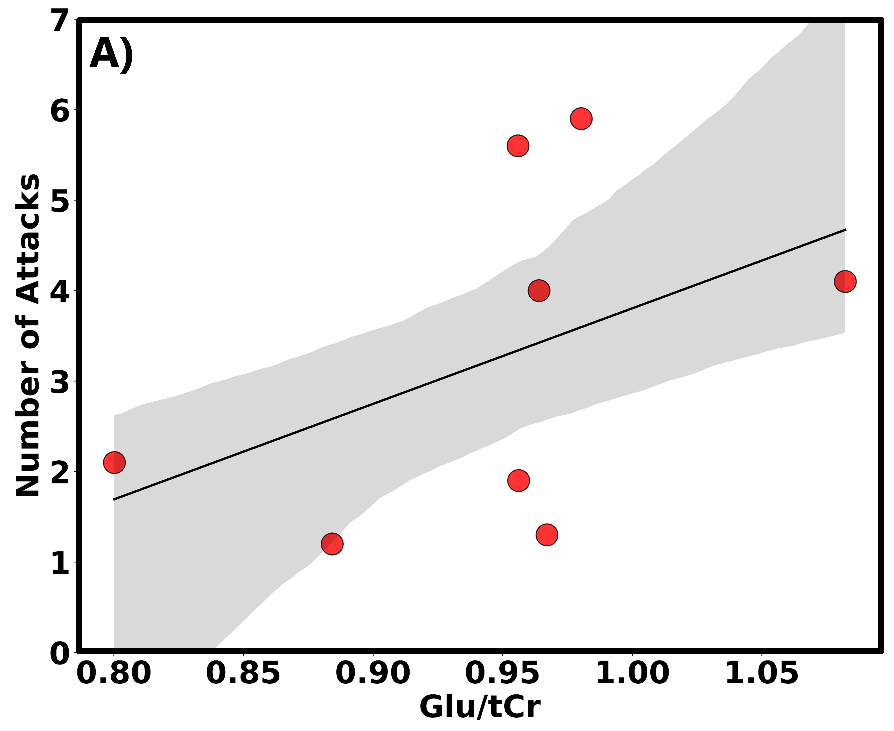

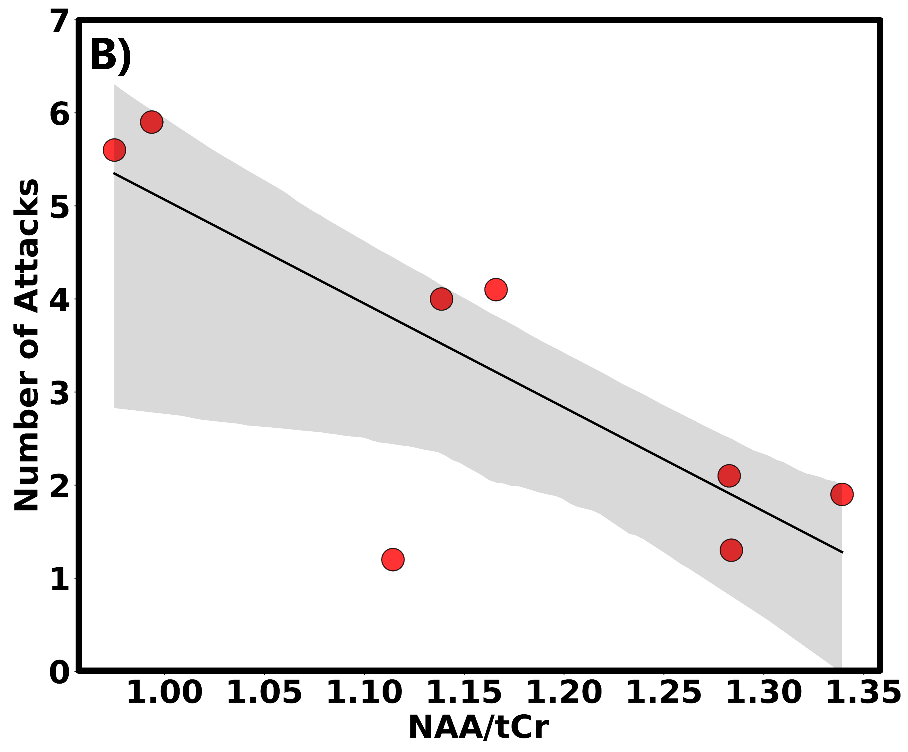


**Figure S1. Distribution of Glu/tCr and NAA/tCr according to attack frequency in cCH patients.**Scatter plots for Glu/tCr (A) and NAA/tCr (B) as a function of the number of daily attacks in patients. Solid lines indicate linear fits with 95% confidence intervals (shaded area). Ordinary least-squares regression analysis showed no significant association for Glu/tCr (p = 0.26), whereas NAA/tCr showed a significant association (p = 0.018). These plots are provided for descriptive purposes only, as attack frequency was not included in the main statistical model and may represent a potential confounding factor.


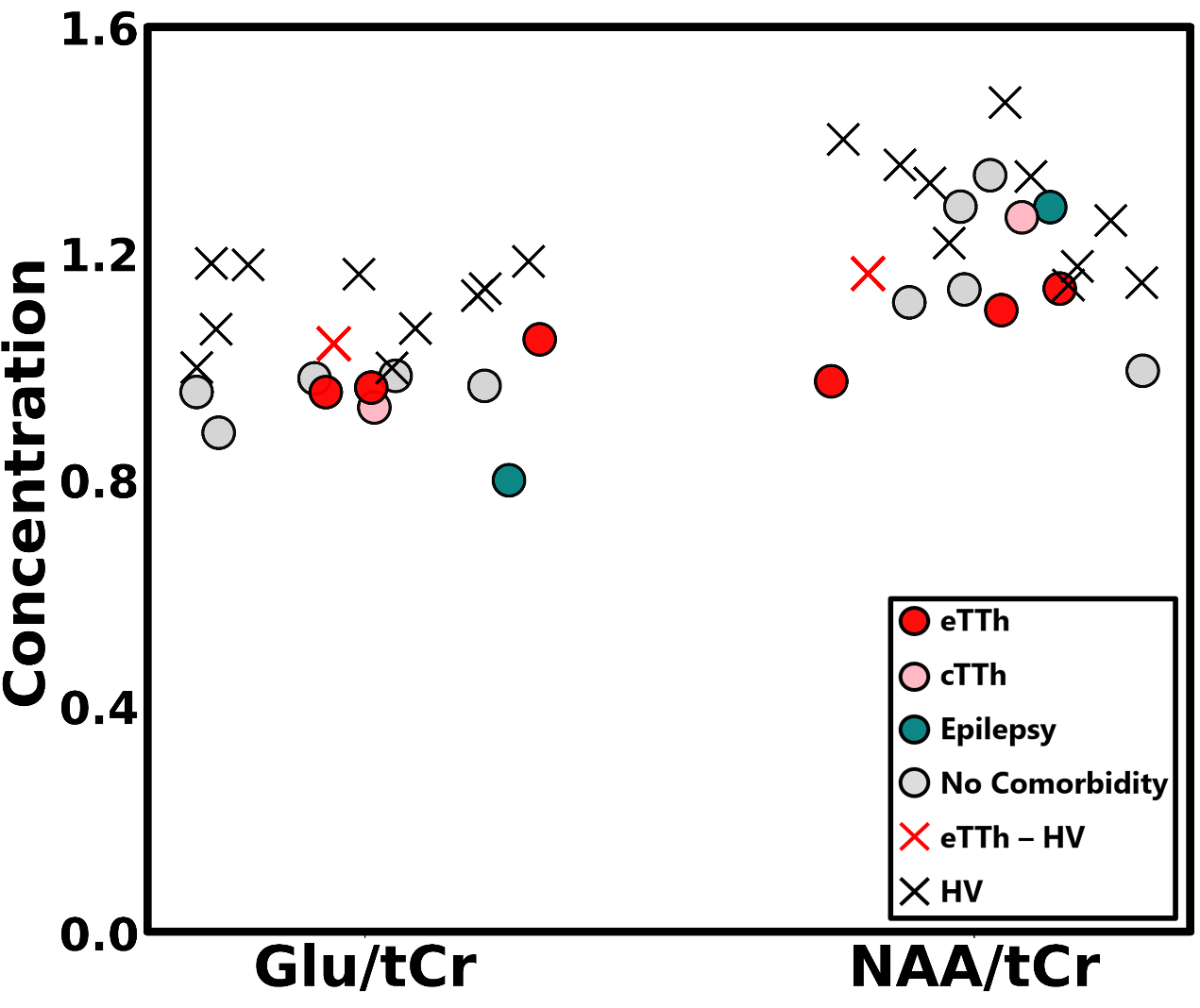


**Figure S2.** **Distribution of Glu/tCr and NAA/tCr according to comorbidities.**
Glu/tCr and NAA/tCr values are shown for individual participants grouped according to the presence of comorbidities, including patients with episodic tension-type headache (eTTH), chronic tension-type headache (cTTH), epilepsy, or no comorbidity. Crosses represent HVs, and red crosses indicate HVs with episodic tension-type headache. These plots are provided for descriptive purposes to illustrate the distribution of metabolite ratios with respect to comorbidities, which may contribute to cohort heterogeneity.


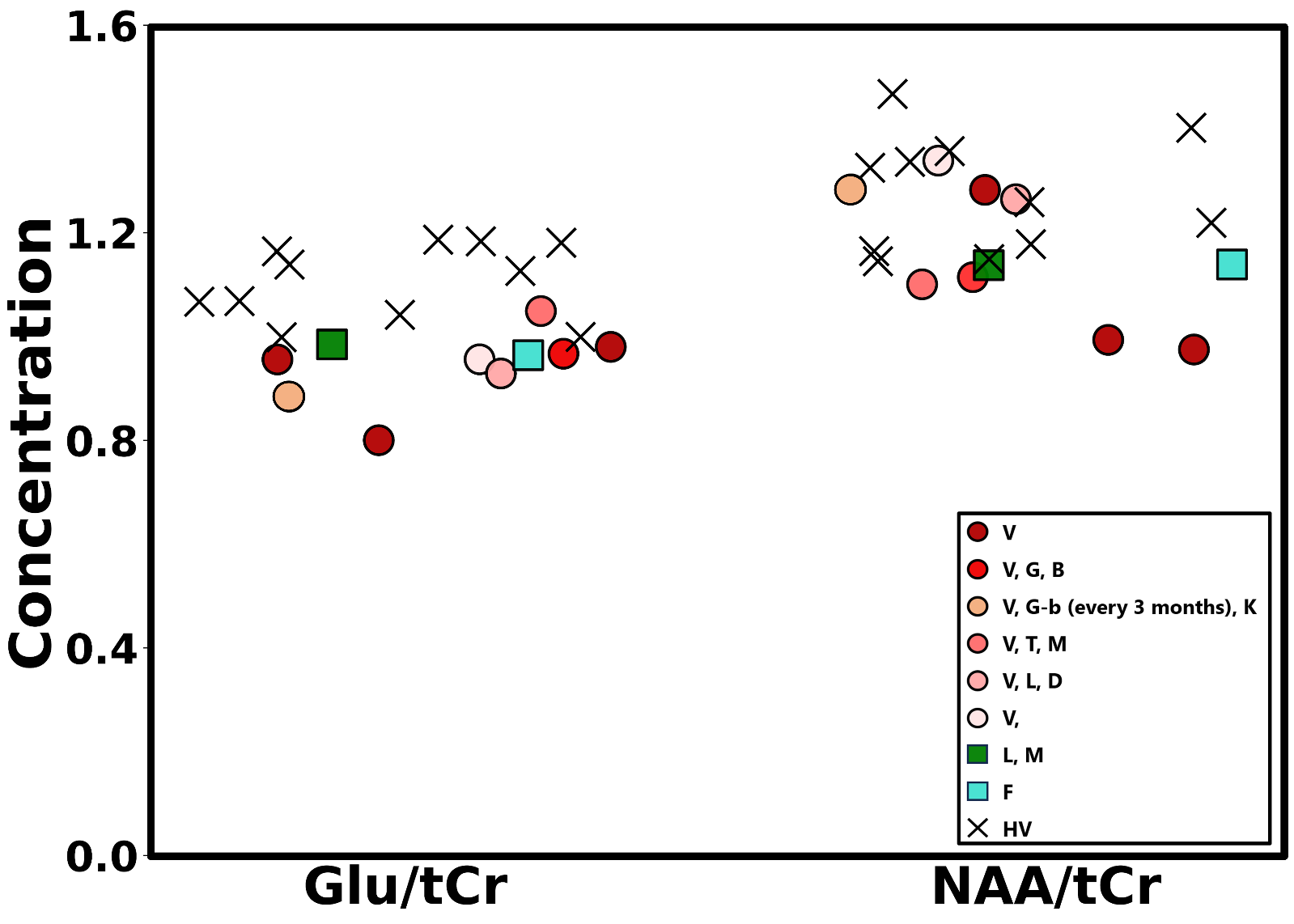


**Figure S3.** **Distribution of Glu/tCr and NAA/tCr according to preventive treatments.** Glu/tCr and NAA/tCr values are shown for individual participants grouped according to preventive medications, as indicated in the legend. All patients treated with verapamil (V) as preventive medication are represented by circles. Crosses represent HVs. These plots are provided for descriptive purposes to illustrate the distribution of metabolite ratios with respect to ongoing treatments, which may represent potential confounding factors. *V = Verapamil, G = Galcanezumab, B = Bupivacaine, G-b = GON-blockade, K = Ketobemidone, T = Topiramate, L = Levetiracetam, M = Melatonin, D = Diazepam, F = Fremanezumab*


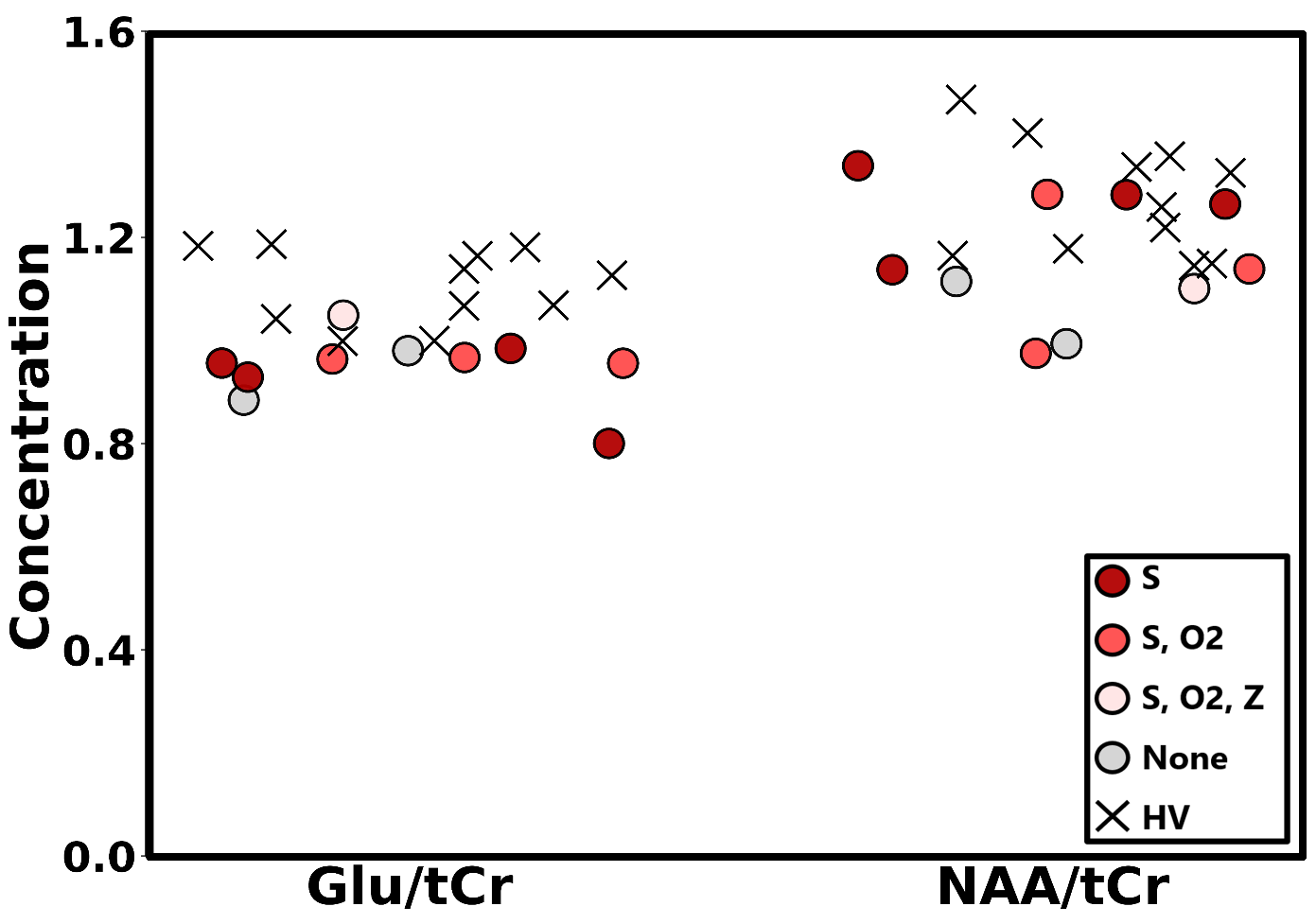


**Figure S4. Distribution of Glu/tCr and NAA/tCr according to acute treatments.** Glu/tCr and NAA/tCr values are shown for individual participants grouped according to acute medications, as indicated in the legend. All patients treated with sumatriptan (S) as preventive medication are represented by circles. Crosses represent HVs. These plots are provided for descriptive purposes to illustrate the distribution of metabolite ratios with respect to possible confounding factors. *S = Sumatriptan, O₂ = Oxygen therapy, Z = Zolmitriptan.*
